# miREM: an expectation-maximization approach for prioritizing miRNAs associated with gene-set

**DOI:** 10.1186/s12859-018-2292-1

**Published:** 2018-08-10

**Authors:** Luqman Hakim Abdul Hadi, Quy Xiao Xuan Lin, Tri Tran Minh, Marie Loh, Hong Kiat Ng, Agus Salim, Richie Soong, Touati Benoukraf

**Affiliations:** 1 Cancer Science Institute of Singapore, National University of Singapore, 14 Medical Dr, Singapore, 117599 Singapore; 2 Translational Laboratory in Genetic Medicine, Agency for Science, Technology and Research, Singapore, Singapore; 3 Department of Mathematics and Statistics, School of Engineering and Mathematical Sciences, La Trobe University, Bundoora, Victoria Australia; 4 Department of Pathology, National University of Singapore, Singapore, Singapore

**Keywords:** Gene regulation, miRNA, Expectation-maximization

## Abstract

**Background:**

The knowledge of miRNAs regulating the expression of sets of mRNAs has led to novel insights into numerous and diverse cellular mechanisms. While a single miRNA may regulate many genes, one gene can be regulated by multiple miRNAs, presenting a complex relationship to model for accurate predictions.

**Results:**

Here, we introduce miREM, a program that couples an expectation-maximization (EM) algorithm to the common approach of hypergeometric probability (HP), which improves the prediction and prioritization of miRNAs from gene-sets of interest. miREM has been made available through a web-server (https://bioinfo-csi.nus.edu.sg/mirem2/) that can be accessed through an intuitive graphical user interface. The program incorporates a large compendium of human/mouse miRNA-target prediction databases to enhance prediction. Users may upload their genes of interest in various formats as an input and select whether to consider non-conserved miRNAs, amongst filtering options. Results are reported in a rich graphical interface that allows users to: (i) prioritize predicted miRNAs through a scatterplot of HP *p*-values and EM scores; (ii) visualize the predicted miRNAs and corresponding genes through a heatmap; and (iii) identify and filter homologous or duplicated predictions by clustering them according to their seed sequences.

**Conclusion:**

We tested miREM using RNAseq datasets from two single “spiked” knock-in miRNA experiments and two double knock-out miRNA experiments. miREM predicted these manipulated miRNAs as having high EM scores from the gene set signatures (i.e. top predictions for single knock-in and double knock-out miRNA experiments). Finally, we have demonstrated that miREM predictions are either similar or better than results provided by existing programs.

**Electronic supplementary material:**

The online version of this article (10.1186/s12859-018-2292-1) contains supplementary material, which is available to authorized users.

## Background

microRNAs (miRNAs) are important modulators of gene expression in various biological systems, including development [1], carcinogenesis [2] and virus-host crosstalk [3]. Most mRNAs can be repressed by more than one miRNA and conversely, most miRNAs have many known and predicted mRNA targets. This direct impact of miRNAs on a large number of mRNA species makes it a powerful biological regulator and therefore a prime candidate for studying diseases such as cancer, where miRNAs can act either as oncogenes (oncomiRs) or tumour-suppressors [4]. Gene repression by miRNAs is generally achieved by pairing between the 5’ end of miRNAs, from the second to the seventh nucleotide (called the seed region), and the 3’ untranslated region (UTR) of the gene target [5]. The strong effects of miRNAs on mRNAs regulation could, in theory, mean that specific miRNA-signatures are linked to certain gene expression patterns. Several databases predicting miRNA’s targets based on various algorithms have been launched. Although the lack of specificity in miRNA’s target predictions is a known fact [6], by taking advantage of these existing miRNA-target databases, numerous programs succeeded in highlighting the most relevant miRNAs affecting a gene expression pattern [7–10]. Indeed, since a miRNA can target several mRNAs, it is possible to compute the statistical significance of the overrepresentation of miRNA’s targets within a gene-set. Most of the aforementioned programs use a standard hypergeometric probability (HP) to predict miRNAs involved in a biological process based on a compendium of miRNA-target databases. This strategy is in line with other tools used to compute the significance of functional annotations from gene-lists. However, when applied to searching miRNA-signatures from gene-lists, there is a considerable overlapping of gene targets between miRNAs leading to a number of significant signatures, including numerous potential false positive predictions [6, 11]. The source of false positives may result from miRNAs sharing a similar seed region. Consequently, it is important that programs and algorithms used are able to highlight the true positives. In contrast to current methods based on HP only, we introduce a novel strategy in complement to HP, which (i) ’weigh-down’ the contribution from overlapping target genes when calculating the significance of each miRNA-signature using an expectation-maximization (EM) algorithm, a general probabilistic framework that can be used for this purpose [12]; and (ii) cluster all predicted miRNAs according to their seed region sequences for identifying “synonymous” predictions. To increase the specificity of our prediction, we also build a large compendium of miRNA’s target predictions based on the most used databases. To our knowledge, the application of EM-algorithm as a probability measurement for significance in functional annotation tools has yet to be explored.

## Implementation

### miREM workflow

**Fig. 1:**
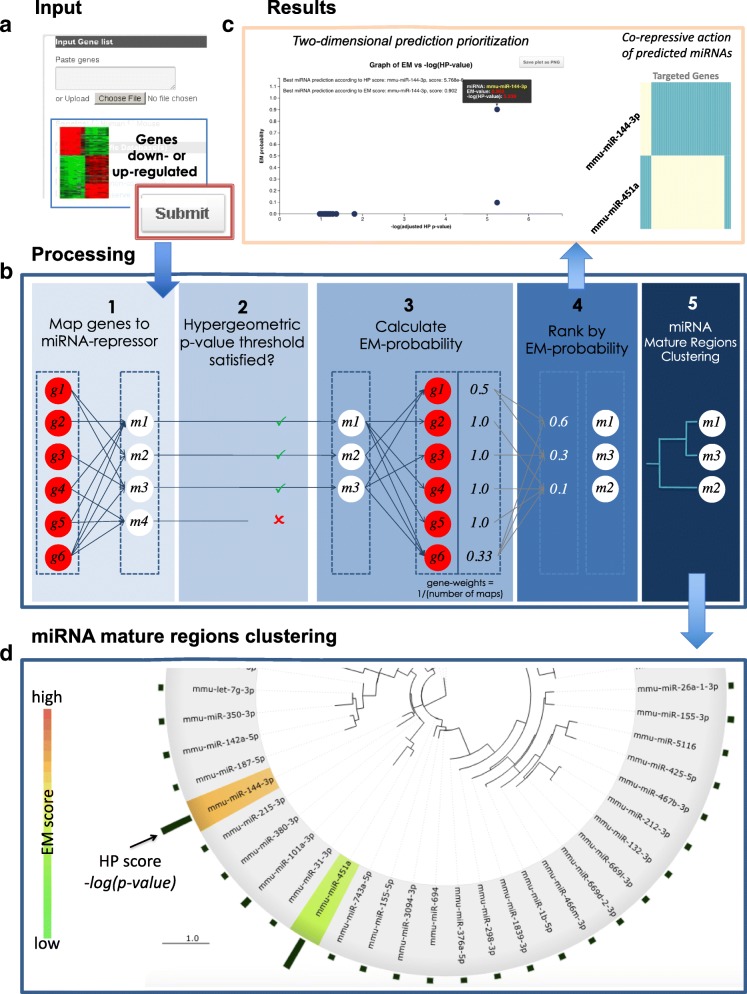
miREM Workflow. **a** The gene-list and setup parameters are entered into the input page. **b** Each transcript is associated to its targeted miRNA(s) using the selected prediction databases. miRNAs are selected only if their respective HP *p*-values reach a pre-defined enrichment level. Then, these selected miRNAs are subjected to the EM-algorithm to establish the likelihood probability of each miRNA. miRNAs with the highest likelihood probabilities are the most likely to have an influence on the DEG. Afterwards, predicted miRNAs are clustered according to their seed region sequences in order to identify duplicated predictions. **c** Subsequently, results are displayed in a dynamic graphical interface allowing an easy data interpretation. **d** Finally, a dendrogram of miRNA seed sequences is generated to help in identifying duplicated predictions (miRNAs sharing similar sequences)

The miREM workflow is composed of the following five steps (Fig. 1):

1. miREM takes a list of differentially expressed genes (DEG) derived from either RNA-seq or the latest microarray platforms as input. Since the input DEG list is obtained from whole transcriptome level, miREM is inapplicable to the results derived from targeted sequencing technologies or microarray platforms targeting a specific gene panel. After receiving a gene list, miREM associates each transcript to its targeted miRNA(s) using the selected prediction databases, thus to create a list of potentially repressive miRNAs.
2. The hypergeometric *p*-value (and corrected *p*-value according to Benjamini-Hochberg) is determined for each unique miRNA so as to identify its enrichment significance.
3. If only one miRNA is found to have a significant *p*-value, the program stops and identifies only this significant miRNA as having an influence on the DEG.
4. If more than one miRNA can be identified, the program then selects miRNAs with corrected *p*-values below the specified threshold and subjects them to the EM-algorithm to establish the likelihood probability of each miRNA. miRNAs with the highest likelihood probabilities are most likely to have an influence on the DEG. Along with tab-delimited files, miREM results are available through a clustered heatmap of miRNA-gene interactions to ease visualization of the genes targeted by predicted miRNAs. This visualization also enables users to intuitively infer the kind of co-repression activity these predicted miRNAs play in the system.
5. Finally, predicted miRNAs are clustered according to their seed sequences in order to identify duplicated predictions (miRNAs sharing similar sequences).

This unique workflow, coupled with a rich combination of features (Table 1), makes miREM a better alternative when compared to existing softwares.

**Table 1:** Feature comparisons of five miRNA predicting tools

	miREM	CORNA	Geneset2miRNA	ChemiRs	Sylamer
Platform	Web-based	R package	Web-based	Web-based	Web-based
					standalone
Installation required	No	Yes	No	No	Optional
GUI	Yes	No	Yes	Yes	Yes
Software last update	2017	2013	2009	2015	Unspecified
Organisms supported	Human, mouse	22 species including	6 species including	Human	7 species including
		human and mouse	human and mouse		human and mouse
Reference databases	Diana,	Miranda	mirDB,	Diana,	Unspecified
	Miranda,		Pictar,	Miranda,	
	mirDB,		PITA,	mirDB,	
	Pictar,		TargetScan	miRWalk,	
	PITA,			RNA22,	
	RNA22,			RNAhybrid,	
	TargetScan			Pictar(4way),	
				Pictar(5way),	
				PITA,	
				TargetScan	
Mirbase version	21 (June 2014)	10.0 (August 2007)	11 (April 2008)	21 (June 2014)	Unspecified
Ability to specify	Yes	No	No	Yes	No
database(s)					
Database last updated	2017	2007	2008	2015	Unspecified
Cross-database query	Yes	No	No	Yes	No
Conserved/ nonconserved	Yes	No	No	No	No
miRNA					
distinction					
Input format	List of DEG:	List of DEG:	List of DEG:	List of DEG:	Full gene list
	text file,	Ensembl	text file,	text file,	ranked by fold
	Gene Symbol,		Gene Symbol,	Gene Symbol,	change
	RefSeq,		RefSeq Protein ID,	Ensembl	
	Ensembl,		RefSeq Transcript ID,		
	UCSC		Ensembl,		
			UniGene,		
			Entrez Gene ID,		
			UniProt/Swiss-Prot,		
Output formats	Tab-delimited file	R list	CSV	CSV	jnlp
	Scatterplot				
	Heatmap				
	Phylogenetic miRNA				
	classification				
Show target gene list	Yes	No	Yes	No	No
Algorithm	Hypergeometric	Hypergeometric	Hypergeometirc	Hypergeometirc	Hypergeometirc
	Expectation	Fisher’s exact test			
	Maximization				
		Chi-square			
Detection of	Yes	No	No	No	No
duplicated predictions					

### miREM’s miRNA-target interactions database

Multiple features characterized in the miRNA-target interaction mechanism have been exploited for developing strategies to predict miRNA target genes, including (i) the seed complementarity between miRNA and mRNA strands; (ii) the free energy of the miRNA:mRNA duplex; (iii) the target site accessibility; (iv) the contribution of multiple binding sites and (v) the evolutionary conservation [13]. Many databases exploit one or several prediction strategies to provide miRNA-mRNA interaction information. In order to build a comprehensive resource to predict a miRNA-signature, we compiled those up-to-date information pooled from the latest releases of Diana [14], Miranda [15], mirDB [16], Pictar [17], PITA [18], RNA22 [19] and TargetScan [20] (Additional file 1: Table S1).

### Querying miREM’s database

miREM accepts Refseq, Ensembl, UCSC IDs or official gene names as input and uses Ensembl Biomart [21] to unify gene IDs. The miREM analysis interface allows interrogation of the reference databases in a flexible way, enabling users to select one or more preferred reference databases. If more than one database is selected, the user can opt to use only the miRNA-target interactions that are found to be common to all (intersections). Alternatively, users can select miRNA-target interactions that appear in one (union of all databases) or more of the seven databases (intersections from 2 to 7 -all- reference databases). Moreover, for Targetscan and Miranda reference databases, queries can be restricted to evolutionary conserved miRNAs. Reference databases are complementary as a large portion of predicted targets are unique to each database (Additional file 2: Figure S1). The flexibility in querying reference databases provides users a leverage to adjust for prediction specificity and sensitivity.

### EM algorithm formulation

Assuming we have *N* genes and *K* miRNAs, let us define an *N*×*K* matrix *Z*, where *z*_*ik*_=1 if gene *i* is repressed by miRNA *k*; otherwise, *z*_*ik*_=0. We are interested in estimating the proportion of genes repressed by the *k*^*th*^ miRNA, *p*_*k*_,*k*=1,…,*K*. By observing matrix *Z*, in other words, we are certain which of the genes are repressed for each miRNA, then the probabilities *p*_*k*_’s can be estimated by counting the number of genes repressed by the miRNA and dividing this by the total number of genes,

1 $$\begin{array}{*{20}l} \hat{p_{k}} = \frac{\sum^{N}_{i=1}z_{ik}}{N} \end{array} $$

However, in reality, we do not directly observe *Z*. Instead, through miRNA-target prediction databases, we observe matrix *Y*=(*y*_*ik*_),*i*=1,…,*N*;*k*=1,…,*K*, where *y*_*ik*_=1 if the *k*^*th*^ miRNA is predicted to target the *i*^*th*^ gene, otherwise *y*_*ik*_=0. We indicate the parameter of interest *θ* as (*p*_1_,…,*p*_*K*_). The likelihood of the complete data (*Y,Z*) can be written as,

2 $$\begin{array}{*{20}l} L(\theta|Y,Z)= \prod_{i=1}^{N}\prod_{k=1}^{K} \left(p_{k}^{z_{ik}}(1-p_{k})^{1-z_{ik}}\right)^{y_{ik}} \end{array} $$

Note that the above likelihood function implicates a logical assumption that *P*(*z*_*ik*_=0∣*y*_*ik*_=0), i.e., gene *i* cannot be targeted by miRNA *k* if the prediction database does not predict this.

The log-likelihood of the complete data is taken as the logarithm of the likelihood and is given by,

3 $$\begin{array}{*{20}l} \ell(\theta|Y,Z)= \sum_{i=1}^{N}\sum_{k=1}^{K} y_{ik}\left[z_{ik} \log p_{k} + (1-z_{ik})\log(1-p_{k})\right] \end{array} $$

To estimate *θ*, we will use EM algorithm that consists of two iterative steps: an E-step where we evaluate the expected value of complete data log-likelihood, *Q*(*θ*|*Y,Z*)=*E*[ *ℓ*(*θ*|*Y*,*Z*)]; and an M-step where we update the parameter estimates using a current estimate *Q*(*θ*|*Y,Z*). The algorithm starts from an initial estimate $\theta ^{(0)}=\left (p_{k}^{(0)},k=1,\dots,K\right)$, where $p_{k}^{(0)}=\frac {1}{K}$. In iteration *m*, we update *θ*^(*m*)^ in two steps:

- E-step: We update the current estimate of matrix *Z* as,
  4 $$\begin{array}{*{20}l} \hat z_{ik}^{(m)} = E\left[z_{ik} | Y_{i},\theta^{(m-1)}\right] = \Pr\left(z_{ik}=1|Y_{i},\theta^{(m-1)}\right) \end{array} $$
  5 $$\begin{array}{*{20}l} = \frac{y_{ik} p_{k}^{(m-1)}}{\sum_{k=1}^{K} y_{ik} p_{k}^{(m-1)} }, \forall i,k \end{array} $$
- M-step: We update the estimate of *θ*, $\hat \theta ^{m}$ by updating each of its component,
  6 $$\begin{array}{*{20}l} \hat p_{k}^{(m)} = \frac{\sum_{i=1}^{N} \hat z_{ik}^{m} }{N}, \forall k \end{array} $$

Intuitively, in the E-step, each *k*^*th*^ miRNA with *y*_*ik*_=1 is assigned a fraction of gene *i* in proportion to $p_{k}^{(m-1)}$, and this fraction is $\hat z_{ik}^{(m)}$ while the M-step updates the probability of each miRNA by averaging the number of genes that are predicted to be repressed by the miRNAs. The algorithm reaches convergence in a few iterations and return the parameter estimates $\hat p_{k}$, which is used to calculate the EM scores.

### Using HP as a filtering step for EM-algorithm inputs

A non-negligible constraint of the EM algorithm is the running-time performance. Indeed, this algorithm is based on multiple iterations with a linear complexity. Therefore, its performance decreases with increments in the input data. Hence, we attempted to limit the number of miRNAs involved in the computation to those that are more likely to be truly significant. To achieve this, we have implemented HP as an initial filtering criterion. Here, the HP is applied to each miRNA, in order to test whether the number of predicted miRNA’s targets are over-represented within the gene-set. Based on the user HP *p*-value threshold, miREM selects only significant miRNAs and proceeds to the EM step. Where only one miRNA signature is significantly predicted by the HP, no EM is involved.

### Classification of miRNA predictions

miRNAs that share similar seed regions are likely to target similar genes. As such, these miRNAs are likely to be co-predicted. In order to identify duplicated predictions, we have implemented a module to cluster miRNAs according to their levels of homology. This classification is done by multiple alignments of miRNA seed sequences using Muscle [22], followed by the generation of a dendrogram computed by PhyML [23] and displayed by jsPhyloSVG [24].

### Availability and requirements

miREM webportal is freely available and can be accessed online at https://bioinfo-csi.nus.edu.sg/mirem2/.

## Results

We have developed miREM, an HP-EM-based program designed to predict miRNA activities from a gene list. miREM’s web server incorporates a large compendium of human/mouse miRNA-target prediction databases and provides rich output results facilitating prioritization and interpretation of predicted results.

To test miREM performance, we benchmarked miREM predictions against CORNA [7], GeneSet2MiRNA [8], ChemiRs [9], and Sylamer [10] results using several datasets with known miRNA activities. These are detailed in three case studies as follows:

### Case study 1: knock-in miRNA experiments

We used two RNAseq expression datasets from miR-155 and miR-1 knock-in experiments in U2OS cells, respectively [25]. In these experiments, we used a gene-set of repressed genes as input (Additional file 3: Table S2) and ran miREM, CORNA, GeneSet2MiRNA and ChemiRs (Table 2 and Additional file 4: Table S3; for Sylamer, whole gene list ranked by fold change was input). miREM has predicted involving miRNAs correctly, with hsa-miR-155-5p and hsa-miR-1-3p ranked at the first and third positions respectively. Similarly, other four tools, namely CORNA, GeneSet2MiRNA, ChemiRs and Sylamer, showed satisfactory predicting performances whereby both miRNAs involved in the experiments were accurately identified (Table 2 and Additional file 4: Table S3).

**Table 2:** Performance of five miRNA prediction tools using two single miRNA knock-in and one miR-144/451 double knock-out experiments

Input data			Predictions				
Datasets	miRNA	DEG	miREM (based	ChemiRs^c^	GeneSet2MiRNA^c^	CORNA^c^	Sylamer^d^
	involved	a	on EM)^b^				
Cytoplasmic	hsa-miR-155	647	hsa-miR-155-5p	hsa-miR-	HSA-MIR-155	hsa-mir-155	has-miR-155
RNA-seq in	knock in		(1/160)	155-5p	(1/23)	(1/2)	
U2OS cells				(1/118)			
Cytoplasmic	hsa-miR-1	743	hsa-miR-1-3p	hsa-miR-1-	HSA-MIR-1 (1/6)	hsa-mir-1	has-miR-1
RNA-seq in	knock in		(3/9)	3p (1/65)		(2/4)	
U2OS cells							
Microarray in	miR-144/451	396	mmu-miR-144-	Not	mmu-mir-144	mmu-mir-144	mmu-miR-
mice	knock out		3p (1/2)	Applicable^e^	(3/3) *	(2/4) *	144
			mmu-miR-451a				
			(2/2)				

^a^DEG list not applicable for Sylamer where a full gene list ranked by fold change was input

^b^Settings: *p*-value threshold = 0.01, EM convergence parameter = 0.001, common mappings from 3 or more databases and nonconserved miRNAs not included

^c^Settings: *p*-value threshold = 0.01 (for ChemiRs, the minimum number of databases is 5 out of 10)

^d^Ranking number / full prediction result number is not available in Sylamer

^e^Mouse databases are not provided

^*^P-value threshold = 0.05 (no result with *p*-value threshold = 0.01)

### Case study 2: double knock-out miRNA experiment

In addition, we analyzed a microarray dataset derived from a miRNA double knock-out experiment [26]. Up-regulated genes from CD71 ^+^/Ter119 ^+^/FSC ^*high*^ bone marrow cells in miR-144/451 ^−/−^mice in comparison with wild-type controls (Additional file 3: Table S2) were input into miREM, CORNA and GeneSet2MiRNA (ChemiRs was excluded from this test as it provides only human miRNA-target databases; for Sylamer, the input gene list was whole gene list ranked by fold change). miREM ranked mmu-miR-144-3p and mmu-miR-451a in the first and second positions respectively. However, no prediction results were available for CORNA and GeneSet2MiRNA when *p*-value threshold was set as 0.01; even if less stringent *p*-value filtering was applied (*p*-value threshold equals to 0.05), only mmu-miR-144 was predicted for both tools (Table 2 and Additional file 4: Table S3). For Sylamer, only mmu-miR-144 was in the result (Table 2 and Additional file 4: Table S3).

### Case study 3: double knock-out miRNA experiment

Finally, we compared miREM’s prediciton performance in a miR-181a1/181b1 double knock-out experiment [27] with other tools. In miREM, loose criterion in HP filtering step (*p*-value threshold = 0.01) resulted in a long list of miRNA candidates. Hence, a more stringent screening setting was applied in HP step (*p*-value threshold = 0.0001). Again, miREM is the only tool which is able to predict both involving miRNAs, with mmu-miR-181b-5p and mmu-miR-181a-5p ranked in first and fourth positions out of total four predictions respectively. Nonetheless, only mmu-miR-181b was predicted by GeneSet2MiRNA while no results were obtained for CORNA and Sylamer (Table 3 and Additional file 4: Table S3).

**Table 3:** Performance of five miRNA prediction tools using a miR-181a1/b1 double knock-out experiment

Input data			Predictions				
Datasets	miRNA involved	Gene list	miREM (based on EM) *	ChemiRs	GeneSet2MiRNA **	CORNA^#^	Sylamer
			mmu-miR-181a-5p		MMU-MIR-181B		
			(rank 4 out of 4)		(rank 1 out of 9)		
RNA-seq in mice	miR-181a/b knock out	243		Not applicable		No result	No result
			mmu-miR-181b-5p				
			(rank 1 out of 4)				

^*^Settings: *p*-value threshold = 0.0001, EM convergence parameter = 0.001, common mappings from 4 or more databases and non-conserved miRNAs not included

^**^p-value threshold = 0.01

^#^*p*-value threshold = 0.05

### Impact of miRNA databases vs algorithms

Integration of EM algorithm with HP test contributes to miREM’s better prediction performance. In order to test the algorithm implemented in miREM while ruling out database bias, we compared the performances of miREM and CORNA (the rest of the test programs were web-server based and could not be modified) on miR-144/451 double knock-out assay using same miRNA-mRNA interaction database (miRanda mouse conserved miRNA database August 2010 release). miREM ranked miR-144 and miR-451 as first and second predictions respectively in its result, whereas 17 miRNAs were predicted by CORNA, among which miR-144 was ranked first while miR-451 ranked 5th (Additional file 5: Table S4). Hence, introduction of EM into miRNA prediction allowed us to better rank candidate miRNAs. Furthermore, the prediction result by miREM was relatively robust. We tested miREM’s performances using different HP *p*-value thresholds and EM convergence parameters given the down-regulated gene list from hsa-miR-155 knock-in experiment. hsa-miR-155-5p remained the first-ranked candidate in various prediction settings (Additional file 6: Table S5).

## Conclusion

The combination of HP and EM algorithm coupled with a large miRNA-target compendium of databases makes miREM a tool of choice to predict and prioritize miRNAs from a given gene list. Programs like miREM rely on miRNA databases, which can be a source of bias, particularly for uncommon miRNAs processed by the Ago2 endonuclease [28], an alternative mechanism independent to Dicer. Therefore, there is still room for improving target predictions by a better characerization of miRNA targets. Overall, we have demonstrated that miREM’s prediction performance is either similar or better than existing programs such as CORNA, GeneSet2MiRNA, ChemiRs and Sylamer.

Finally, the versatility of the miREM web server makes it accessible to a large panel of users including non-bioinformaticians, by facilitating result exploration and interpretation through numerous representations and a dynamic graphical interface.

## Additional files

Additional file 1 **Table S1.** Release notes of human and mouse miRNA reference databases in miREM. (XLSX 35 kb)

Additional file 2 **Figure S1.** Overlap of miRNA-mRNA predicted interactions across human and mouse reference databases. (PDF 833 kb)

Additional file 3 **Table S2.** List of genes differentially expressed used in the case studies. (XLSX 75 kb)

Additional file 4 **Table S3.** Full predicted results for four tested experiments. (XLSX 514 kb)

Additional file 5 **Table S4.** Predicted results of miREM and CORNA with same database. (XLSX 47 kb)

Additional file 6 **Table S5.** Predicted results of miREM using different HP *p*-values and EM parameters. (XLSX 115 kb)

## Abbreviations

DEG: Differentially expressed genes
EM: Expectation-maximization
HP: Hypergeometric probability
miRNA: Micro RNA
mRNA: Messenger RNA
