# Supplementary material for: miREM: an expectation-maximization approach for prioritizing miRNAs associated with gene-set
Source: BMC Bioinformatics. 2018 Aug 10;19:299. doi: 10.1186/s12859-018-2292-1 (PMC6086043; doi:10.1186/s12859-018-2292-1)
Supplement: Supplementary file 2 — Figure S1. Overlap of miRNA-mRNA predicted interactions across human and mouse reference databases. (PDF 833 kb) [file 12859_2018_2292_MOESM2_ESM.pdf]

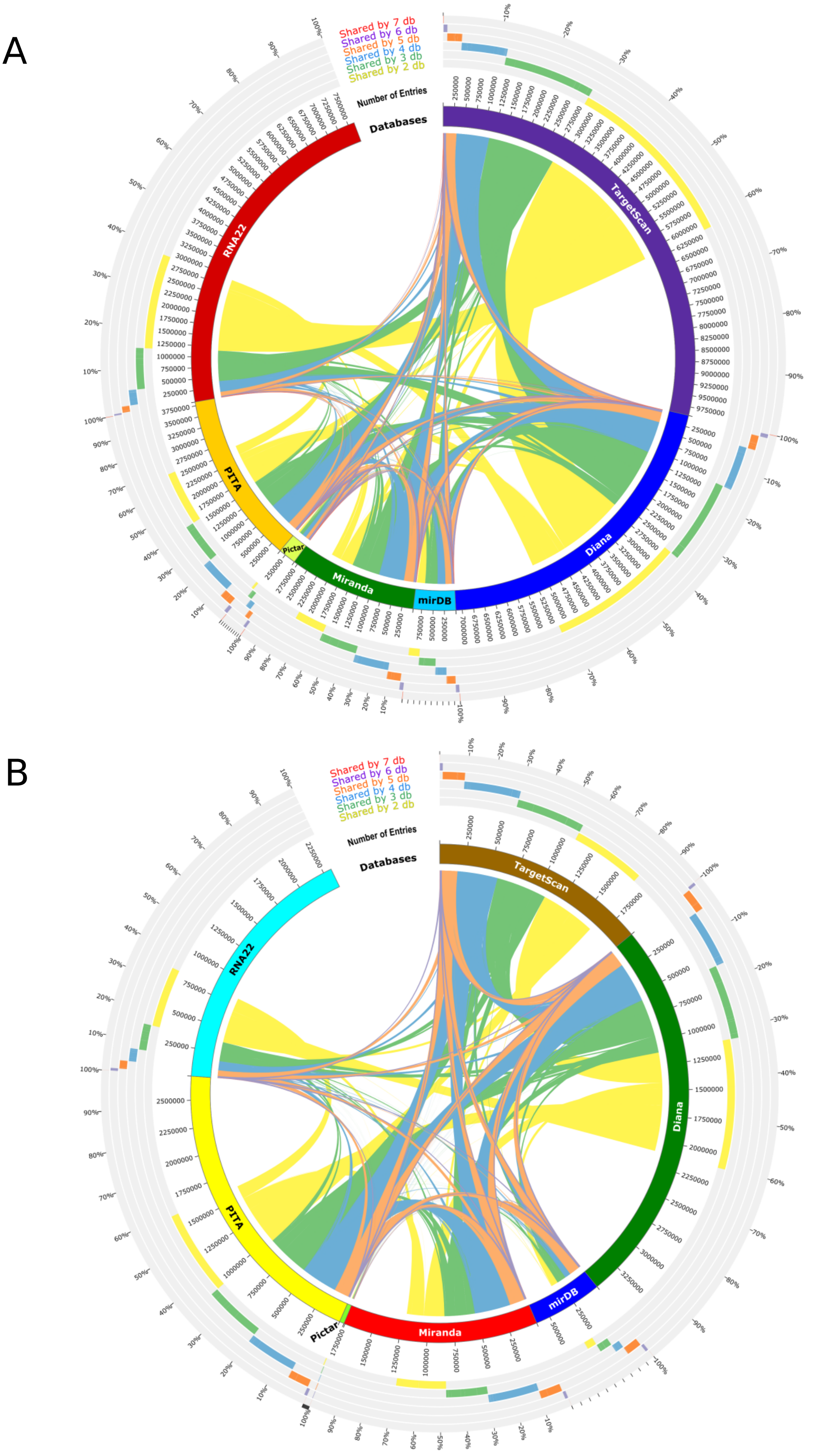

Supplementary Figure 1. Overlap of miRNA-mRNA predicted interactions across human and mouse reference databases. A. miRNA targets shared throughout human miRNA databases. B. miRNA targets shared throughout mouse miRNA databases
